# Bile Acid Signaling in Inflammatory Bowel Disease

**DOI:** 10.3390/ijms22169096

**Published:** 2021-08-23

**Authors:** Mariusz A. Bromke, Małgorzata Krzystek-Korpacka

**Affiliations:** Department of Biochemistry and Immunochemistry, Wroclaw Medical University, Chałubińskiego 10, 50-368 Wrocław, Poland; malgorzata.krzystek-korpacka@umed.wroc.pl

**Keywords:** bile acids, inflammatory bowel disease, signaling, microbiome

## Abstract

Inflammatory bowel disease is a chronic, idiopathic and complex condition, which most often manifests itself in the form of ulcerative colitis or Crohn’s disease. Both forms are associated with dysregulation of the mucosal immune system, compromised intestinal epithelial barrier, and dysbiosis of the gut microbiome. It has been observed for a long time that bile acids are involved in inflammatory disorders, and recent studies show their significant physiological role, reaching far beyond being emulsifiers helping in digestion of lipids. Bile acids are also signaling molecules, which act, among other things, on lipid metabolism and immune responses, through several nuclear and membrane receptors in hepatocytes, enterocytes and cells of the immune system. Gut microbiota homeostasis also seems to be affected, directly and indirectly, by bile acid metabolism and signaling. This review summarizes recent advances in the field of bile acid signaling, studies of inflamed gut microbiome, and the therapeutic potential of bile acids in the context of inflammatory bowel disease.

## 1. Introduction

The objective of this review is to summarize current knowledge of bile acid metabolism and signaling in relation to inflammatory bowel disease. Inflammatory bowel disease (IBD) is a chronic, idiopathic and complex condition of the gastrointestinal tract. Its two most common forms are ulcerative colitis (UC) and Crohn’s disease (CD). The incidence and prevalence of IBD in recent years is on the rise. For illustration, it is expected that in Canada the prevalence rate will increase from 0.5% in 2008, through 0.7% in 2018, up to 1.0% in 2030 [1]. IBD pathogenesis is not fully elucidated but it is widely accepted that genetic, immune, and environmental factors together lead to the disruption of a delicate homeostasis between host immunity and the digestive tract microbiome [2]. To inhibit mucosal immune responses and production of proinflammatory cytokines, therapeutic measures employ anti-inflammatory drugs, including glucocorticoids, immunosuppressants, aminosalicylates, and biologics [3]. This review describes the action of bile acids and their receptors on intestinal immune system and on intestinal microbiota, which could be taken into consideration as potential therapeutic targets in IBD.

A link between inflammatory gastrointestinal disorders and bile acids has been known for a long time [4,5,6]. Vantrappen et al. demonstrated that CD, but not UC patients, have a reduced bile acid pool size when compared to normal subjects. The decrease in bile acid concentration was negatively correlated with the Crohn’s Disease Activity Index (CDAI) [5].

Bile is a liver product released into the duodenum to facilitate absorption of lipids or fat-soluble vitamins. Prior to the release into the duodenum, it is collected in the gallbladder. Bile is an aqueous solution of bile acids, bilirubin, lipids (such as cholesterol, free fatty acids and phosphatidylcholine), enzymes and proteins, amino acids as well as inorganic salts [7]. Bile acids (BA) are water-soluble metabolites of cholesterol, and as such can be regarded as lipid molecules. There are two pathways of primary BA synthesis in hepatocytes. The classic pathway, which accounts for more than 90% of total BA production, starts with microsomal cholesterol 7α-hydroxylase (CYP7A1), a rate-limiting enzyme, producing cholic acid (CA) and chenodeoxycholic acid (CDCA) [8]. The other pathway, also known as acidic, is initiated by sterol-27-hydroxylase (CYP27A1) and predominantly yields CDCA. In the liver, CA and CDCA are further metabolized by conjugation with glycine and taurine. Conjugation changes BA into stronger acids, which limits their passive reabsorption as BA, pass down the biliary tree. In humans, the conjugation with glycine is prevalent and accounts for ≈90% of the BA pool. After being released into the intestinal lumen, bacteria can produce secondary bile acids. The main BA modifications in the human large intestine include deconjugation, oxidation of hydroxyl groups at C-3, C-7, and C-12, and 7α/β-dehydroxylation. Dehydroxylation of cholic acid leads to formation of deoxycholic acid (DCA), while CDCA is metabolized to lithocholic acid (LCA), a monohydroxy bile acid. In addition, the deconjugation is the result of bacterial metabolism, in which glycine and taurine are released from BA. Conjugated bile salts and deconjugated BA are reabsorbed in the intestine and through enterohepatic circulation return to the liver to refill the BA pool [9]. BA uptake into enterocytes occurs principally in the terminal ileum via the apical sodium-dependent bile acid transporter (ASBT) [10]. Approximately 400–800 mg of bile salts escapes the enterohepatic circulation daily and becomes a substrate for significant microbial biotransforming reactions in the large bowel [9]. Table 1 summarizes the extent of microbial metabolism of BA as they pass through the intestines.

Colonic epithelium forms not only a physical boundary between the complex environment of colon lumen and the host tissues. There are other elements, which can be regarded as components of the intestinal barrier such as outer mucus layer, luminal microbiota colonizing it is surface, and inner subepithelial elements, such as T cells, B cells, eosinophils, mast cells, dendritic cells, and macrophages [12]. In IBD, the functioning of the intestinal barrier is disrupted leading to permeability defects. Altered tight junction structure, protein composition and their functioning were observed in IBD patients [13]. Moreover, intestinal permeability test results could be used to formulate a prognosis of clinical relapse in patients with IBD in remission [14]. In turn, these barrier defects allow entry for luminal microbes and increase influx of toxins and allergens, which further promote immune cell infiltration into the gut tissue and an inflammatory response.

## 2. Bile Acid Regulation and Signaling

One of the main traits of the BA is the possession of a hydrophobic and a hydrophilic surface, which makes these molecules water-soluble and gives them detergent properties. The strength of BA detergent action greatly depends on the distribution and orientation of hydroxyl groups around the steroid backbone, and it follows the order: LCA > DCA > CDCA > CA > UDCA [11]. Cytotoxicity of BA also follows this distribution [10]. However, next to the role in the uptake of nutrients, BA can act as regulatory molecules as well. Bile acids interact with nuclear farnesoid X receptor (FXR), and the degree of the interaction depends on structural modifications of a bile acid-CDCA is the most potent FXR agonist, followed by DCA, LCA, and lastly, CA [8]. Beyond FXR, BA binds with other nuclear receptors such as pregnane X receptor (PXR), constitutive androstane receptor (CAR), vitamin D receptor (VDR), and small heterodimer partner (SHP), which, in turn, is regulated by the FXR and constitutes an integral part of the BA signaling [8]. Intestinal epithelial cells express PXR that regulate the pattern of cytokines and chemokines produced. The activation of PXR increases tumor growth factor-β (TGF-β) and interferon γ-induced protein 10 kDa (IP-10) expression, while it also reduces the expression of tumor necrosis factor-α (TNF-α), interleukin 8 (IL-8), and C-C motif chemokine ligand 5 (CCL5, also known as RANTES) [15]. In the liver and intestine, FXR maintains the BA homeostasis by regulating the transcription of genes involved in BA synthesis, absorption, and export. FXR inhibits the BA synthesis through a feedback mechanism, mainly by reducing the expression of the rate limiting enzyme of the bile acid synthesis, namely, microsomal cholesterol 7α-hydroxylase (CYP7A1) [16]. FXR upregulates hepatocyte apical bile acid efflux transporters, whichpromotes the secretion of bile acids into the gallbladder and prevents toxic effects of over-accumulation of BA in the liver cells. These transporters include bile salt export pump (BSEP), multidrug resistance protein 2 (MDR2) and MDR3, and multidrug resistance-associated protein 2 (MRP2) [8]. BA are also perceived as signal molecules by the G-protein bile acid-activated receptor-1, widely known as TGR5 (Takeda G-coupled receptor 5). BA interact with TGR5 with different strength: LCA > DCA > CDCA > UDCA > CA. In mice, taurine- and glycine-conjugated BA are also agonists of the hepatic sphingosine 1-phosphate receptor 2 (S1PR2) [17]. In contrast to S1PR2, TGR5 recognizes BA regardless of their substitution and conjugation state [18]. Secondary oxo-bile acid, 3-oxoLCA, inhibits Th17 cell differentiation by physically interacting with retinoid-related orphan receptor-γt (RORγt), and inhibiting its transcriptional activity [19]. In studies on mice, secondary BA (e.g., LCA/3-oxo-LCA) modulate colonic FOXP3^+^Treg cells expressing RORγt through the vitamin D receptor and ameliorate host susceptibility to colitis [20].

**Figure 1:**
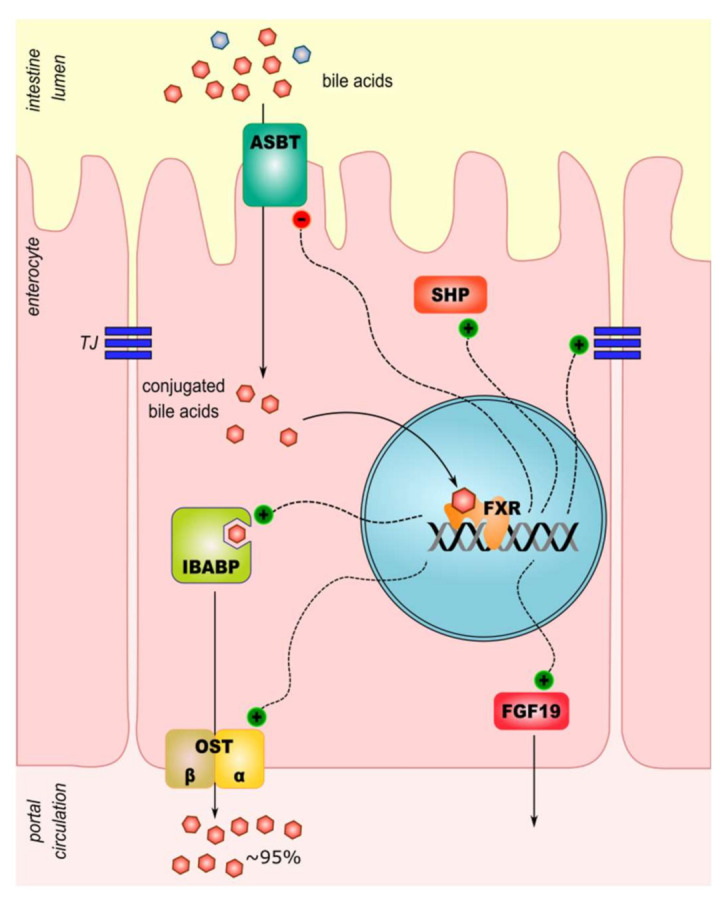
A model of intestinal cellular transport and signaling of bile acids. A majority of conjugated bile acids is reabsorbed by the intestinal epithelial cells. Unconjugated bile acids escape uptake through ASBT and enter the colon where they undergo further metabolism by local microbiota to generate secondary bile acids. ASBT, sodium-dependent bile acid transporter; FGF19, fibroblast growth factor 19; FXR, farnesoid X receptor; IBABP, ileal bile acid-binding protein; OSTα/β, organic solute transporter α/β; SHP, small heterodimer partner.

The FXR expression is the highest in the liver, but in the intestine, it plays an important role in the bile acid metabolism, too. FXR activation reduces expression of apical sodium-dependent bile acids transporter (ASBT) [8]. Next, the export of BA from enterocytes is also activated by FXR. The expression of the ileal bile acid-binding protein (IBABP) and the heterodimeric organic solute transporter alpha and beta (OSTα/β) increases through the action of activated FXR [8]. In intestinal mucosa, cells and macrophage FXR exert anti-inflammatory activity. It modulates other signaling pathways by inhibition of targets of pro-inflammatory activator protein-1 (AP-1) and nuclear factor-kappa B (NF-*κ*B): tumor necrosis factors-*α* (TNF-*α*), IL-1, IL-6, cyclooxygenase-1 (COX-1), COX-2 [21]. Sensing intestinal microbiota and regulation of the innate immune response are other signaling pathways in which FXR may play a role. Following intracellular toll-like receptor 9 (TLR-9) activation, interferon-7 (IRF-7) physically interacts with the FXR promoter and drives its expression. Interestingly, activation of membrane-localized TLR-4 in macrophages downregulates the expression of FXR [22]. A summary of BA transport and signaling in an enterocyte is presented in Figure 1.

The FXR functions as a negative modulator of an assembly of NOD-, LRR- and pyrin domain-containing protein 3 (NLRP3) inflammasome through a physical interaction with NLRP3 and caspase 1. Moreover, the SHP is a negative regulator of NLRP3 inflammasome activation and an intrinsic negative regulator of TLR-triggered inflammatory responses [23,24].

Fibroblast growth factor 19 (FGF19) is the key enterokine produced in response to intestinal FXR activation. The FGF19 is transported to the liver where it binds to the FGF-R4/βklotho complex on hepatocytes membranes. One of the targets of this signaling is the key enzyme of bile acids synthesis, CYP7A1; repression of its activity in response to FGF19 lowers bile acid synthesis in hepatocytes [25]. In an interesting experiment, mice lacking FXR had an upregulated BA synthesis and experienced bacterial overgrowth, increased intestinal permeability and a high rate of bacterial translocation to mesenteric lymph nodes. This phenotype could be rescued by adenoviral-mediated expression of modified (non-tumorigenic) human FGF19 [26]. Moreover, FGF-19 concentration in blood of healthy controls and CD patients with active inflammation or in remission has been compared. Mean concentration of the enterokine was lower in CD patients [26]. The therapeutic potential of FGF-19 analogues in context of intestinal inflammation in primary sclerosing cholangitis (PSC) is confirmed by recently completed clinical trials [27,28].

**Figure 2:**
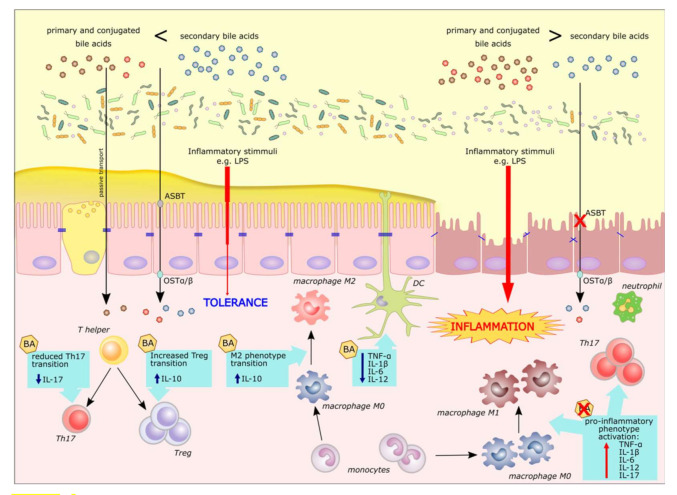
Regulatory role of bile acids in intestinal inflammation. Bile acids in the intestine are actively transported into lamina propria through sodium-dependent bile acid transporter (ASBT) and a complex of organic solute transporters α and β (OSTα/β) or by passive absorption. Bile acid signaling suppresses the pro-inflammatory phenotype of immune cells through reduced release of TNF-α, IL-1β, IL-6 or IL-12. It also stimulates the production of an anti-inflammatory IL-10 cytokine and epithelial barrier renewal (not shown). Inflammatory bowel disease patients experience reduced re-absorption of bile acids as well as disturbed intestinal bile acid metabolism by changed (reduced diversity) microbial community. A breach of the epithelial barrier may further exacerbate inflammation.

The membrane receptor for bile acids, TGR5, belongs to a family of G-protein coupled receptors. The receptor is expressed in the distal ileum and colon, cells of the biliary tracts, immune cells and colon-innervating neurons. In contrast to FXR, TGR5 is not expressed by hepatocytes [25]. One of its functions is the regulation of gastrointestinal motility in response to BA signals. Deoxycholic acid, lithocholic acid, or oleanolic acid (a selective agonist of TGR5) stimulated colonic motility in mice [29]. Animal and human studies have shown the role of the receptor in modulating intestinal inflammation. Biagioli et al. demonstrated that treating mice with a semi-synthetic BA in murine models of colitis, reduces the level of intestinal inflammation. Inhibition of expression of proinflammatory cytokines and chemokines (IL-1β, IL-6, IFN-γ, TNF-α and CCL2) was observed in treated animals. Moreover, the TGR5 agonist induced expression of anti-inflammatory genes (IL-10 and TGF-β). The authors speculate that increased expression of IL-10 in the colon after the treatment, exposes monocytes and lymphocytes to this anti-inflammatory cytokine, which is likely responsible for shifting macrophages from an M1 pro-inflammatory to an M2 anti-inflammatory phenotype [30]. Inflammation seemed to sensitize the colon to BA signaling as colonic expression of TGR5 mRNA increased in rodent models of colitis. Moreover, an elevated expression of the mRNA was observed in the colon of Crohn’s disease patients compared to non-inflamed colons [31]. Further associations of TGR5 with IBD have been observed in studies on the primary sclerosing cholangitis and PSC-related UC. In a study with sequencing of TGR5 from PSC patients and healthy controls, several nonsynonymous variants of TGR5 have been found. These mutations abolished or reduced TGR5 activity, but were too rare to explain the statistical associations. Furthermore, a fine-mapping of the locus at chromosome 2q35 revealed an overall association between the TGR5 single-nucleotide polymorphism rs11554825 and PSC and UC [32]. In another recent genome-wide association study, the TGR5 gene, at the locus 2q35, has been found to be associated with PSC (without information of colitis among studied population) [33]. An illustration of various inflammatory processes in which BA act as signaling molecules is given in Figure 2.

Like FXR, TGR5 also inhibits the activation of the NLPR3 inflammasome. Activation of TGR5 by secondary BA, DCA and LCA, induces the signaling cascade TGR5-cAMP-PKA leading to ubiquitination of NLRP3, thus inhibiting its activation [34].

## 3. Profile of Bile Acids in Ulcerative Colitis

Inflammatory bowel disease is a common comorbidity in patients with primary sclerosing cholangitis. A recentstudy on bile acids content in plasma of PSC patients, included a subset of UC and CD patients. Ulcerative colitis patients had significantly increased fraction of CA and decreased fractions of LCA, DCA and hyodeoxycholic acid. In case of patients with Crohn’s disease and indeterminate IBD, CDCA levels were elevated relative to the non-IBD patients. Ratios of glycine- to taurine-conjugates did not differ based on IBD status. Similar concentrations of BA and similar percentages of conjugates in total BA were observed between PSC patients without IBD and UC patients. Patients with CD had lower concentrations total BA than these groups. Notably, the PSC patients without IBD had significantly elevated CA:DCA ratio compared to controls. In PSC with UC, the CA:DCA ratio was even higher. It has been concluded that the increased fraction of primary BA is an independent phenomenon in PSC that is further exacerbated by IBD [35].

Interesting data were delivered by Quraishi et al., who compared transcriptome of colon wall from PSC patients, UC patients and healthy control subjects [36]. PSC patients often develop colitis, but it is phenotypically different from UC. The gene expression study has shown that colitis types are primarily immune-mediated, with upregulation of pathways of innate, adaptive, and humoral immune responses. Patients with PSC-IBD had very distinct mucosal transcriptomic profile compared with UC alone. Nuclear receptors FXR, pregnane X receptor, and peroxisome proliferator-activated receptor gamma (PPAR-γ) were upregulated in PSC-IBD compared with UC samples. This was also the case for genes coding bile acid transporting proteins: IBABP and multiple basolateral bile acid efflux transporters, including OSTα/OSTβ, BSEP, breast cancer resistance protein (BCRP), and MRP3 [36]. Observed elevated plasma CA:DCA ratio (increased primary BA) might be the metabolic signal inducing measures to limit BA toxicity in PSC complicated with IBD.

The differences in BA concentrations between PSC-IBD and IBD patients (UC and CD together) was a research focus of Torres et al. [37]. The total serum BA pool was two times bigger in PSC-IBD than in IBD patients. Differences in relative content of individual BA were not significant, though CA seem to represent larger fraction of total BA in sera of IBD patients. In fecal samples, median total BA pool was significantly lower in PSC-IBD patients than in IBD patients (167.2 µmol/L vs. 282.4 µmol/L, respectively). The authors reported enrichment, although non-significant, of primary BA in total fecal BA pool in PSC-IBD patients. It is worth noting that no differences in UDCA concentrations were observed between PSC-IBD patients treated with this bile acid and naïve IBD patients [37]. In the metabolomic analysis of plasma BA by Mousa et al. [35], UDCA treatment was regarded as a confounding factor. One should note that studies by Torres et al. [37] and Quraishi et al. [36] were conducted on very small populations (30 participants in all groups in both studies), whereas Mousa et al. recruited 508 patients with PSC and 302 controls [35].

Gene expression analysis of descending colon biopsies from UC patients have shown reduced mRNA levels of BA transporters [10]. This reduction was even stronger in patients with pancolitis. Reduced mRNA expression levels have been observed for the basolateral efflux pumps MRP3, OSTα/β, as well as two apical efflux pumps, MDR1 and MRP4. In patients with UC in remission, quantitative PCR revealed no significant changes in BA transporter mRNA levels compared with those in controls or in patients with active UC [10]. Additionally, Jahnel et al. compared controls with treatment-naïve adolescent UC patients and observed significantly lower FGF-19 mRNA expression [10].

Recent studies suggest that BA are likely to play a role in the enhanced epithelial permeability that is associated with disease progression. Intestinal microbial dysbiosis might be involved in the development of UC. In a study by Baumgartner et al., as much as 34% of UC patients and 22% of CD patients had yellow-green bacterial biofilms in the ileum and right-sided colon. These adherent layers of dense biofilms have been observed in only 6% of controls. A metabolomic analysis of the biofilm have found an accumulation of taurocholic acid that correlated with fecal BA excretion. Moreover, a correlation of mucus production with total amount of BA was observed in UC patients [38].

## 4. Profile of Bile Acids in Crohn’s Disease

The terminal ileum and colon are the most common sites affected in Crohn’s disease, observed in approximately 50% of CD patients. About 30% of patients have only small bowel involvement, and the remaining 20% have isolated colonic involvement [39]. The inflammation of the gut may result in malabsorption of BA and nutrients. Analysis of gene expression in biopsy specimen have revealed significantly lower expression of ASBT and bile acids enterocyte-apical efflux transporter BCRP in patients with CD-associated ileitis than in controls [10]. This seems to lead to BA malabsorption and changes the BA profiles observed in serum and feces of patients with CD. Inflamed mucosa had also lower expression of the sulphating enzyme SULT2A1, a component of the ileal bile acids detoxification system, whereas in CD patients in remission, expression levels of SULT2A1 were equal to those in controls. The FGF19 mRNA expression was significantly lower in treatment-naïve adolescents with CD-associated ileitis. In these patients, mRNA expression levels of ASBT and FXR decreased as well [10]. As demonstrated in vitro and in vivo, human ASBT expression is induced by glucocorticoids. Thus, Jung et al. suggested that induction of ASBT by glucocorticoids could be beneficial in patients with Crohn’s disease who exhibit reduced ASBT expression [40].

The number of possible combination of reactions yielding BA and bile salt is quite large. Thus, it should not be surprising to discover new conjugated bile acids in mice and humans. A recent study reported a discovery of cholic acid conjugated with Phe, Tyr and Leu (phenylalanocholic acid, tyrosocholic acid, and leucocholic acid). The Phe, Tyr, and Leu amino acid conjugates were undetectable upon exposure to antibiotics, suggesting involvement of the microbiome. In the experiments following the discovery, a strain of *Clostridium bolteae* was isolated and it was demonstrated that it could synthesize both Phe-CA and Tyr-CA [41]. The clostridia are known to oxidize, epimerize and deconjugate BA [42]. *C. bolteae* is a bile-resistant human intestinal bacterium, which can cause intra-abdominal infections when the natural intestinal barrier is altered [43]. Interestingly, all three metabolites were significantly higher in the dysbiotic state associated with patients with CD, but not in patients with UC [41]. The role of newly discovered conjugated BA remains to be studied and clarified in the future.

Recently, a very broad study reported MDR1 and BA playing key roles in shaping Teff cell function in the ileum [44]. The study has demonstrated that MDR1 mitigates BA-induced oxidative stress in Teff cells. Upon contact with conjugated BA, Mdr1-depleted Teff cells over-expressed genes encoding proteins involved in the oxidative stress response, DNA damage and nucleotide excision repair, apoptosis and p53 signaling, and NF-κB signaling. Interestingly, in vitro experiments with glycine- or taurine-conjugated bile acids (sampled mice small intestine luminal content and commercially synthesized conjugated BA) inhibited a dye efflux mediated by human MDR1 [44]. Beyond mouse models and in vitro experiments, the authors have found that MDR1 activity was reduced in CD and UC patients relative to healthy controls, but was not different between forms of IBD. Furthermore, the researchers identified MDR1 loss-of-function in five patients with aggressive ileal CD and in three UC patients [44].

Untargeted metabolomics has been applied to study metabolite profile of stool from IBD (CD + UC) patients in comparison to healthy controls [45]. Distinct metabolic profiles for controls and CD patients could be obtained, whereas profiles of UC patients were diffused, what might be explained, at least in part, by subjects’ levels of active inflammation. From the collected data on >8000 metabolic features, decrease in DCA and LCA, as well as the accumulation of primary BA, was characteristic for the active IBD. Sphingolipids, in form of ceramides and sphingomyelins, were also overabundant in stool samples from IBD patients [45]. These lipids are not only components of biological membranes, but act as signaling molecules in cell fate decisions. It has been shown that simple sphingolipids (ceramide-1-phosphate, ceramide and sphingosine-1-phosphate) can function as potent proinflammatory messengers, favoring and exacerbating IBD [46]. Additionally, triacylglycerols were enriched in controls relative to CD and UC subjects [45]. This might reflect the intestinal dysbiosis and changes in bile-related emulsification and uptake of lipids.

Chronic diarrhea that is observed in CD patients might be connected with bile acid malabsorption (BAM) type I, which is a secondary BAM mainly due to ileal dysfunction [47]. This can be explained by the fact, that the distal ileum is responsible for active reabsorption of conjugated BA. Over-accumulated BA in intestine might modulate the structure of tight junctions what in turn might contribute to increased gut permeability [48]. Moreover, BA in jejunum slow down the transit [49], whereas BA in large intestine stimulate motility [29]. Thus, the disturbed BA signaling is most likely responsible for chronic diarrhea experienced by CD patients. In retrospective studies, the prevalence of BAM was very high (between 61% and 93%) [50,51] in CD patients treated with ileal resection. BAM was also present in a substantial number of the CD patients with ileitis or colitis who were not treated by the surgery (14% and 11%, respectively) [50]. BA sequestrants, such as colesevelam and cholestyramine, seem to be effective in treatment of BAM in CD, also post-surgery. In clinical studies, colesevelam treatments were associated with a reduction in watery stool frequency and an improvement in quality of life [52,53].

## 5. Treatment with Bile Acids

Application of BA in order to restore functionality of the intestine is already an established treatment strategy. Ursodeoxycholic acid (UDCA) is approved for clinical use in treatment of, e.g., primary sclerosing cholangitis [35]. It is under clinical trials in other indications such as liver diseases or for administration to patients before a retinal disattachment surgery [54]. The mechanisms of action are not well defined, but it is believed that UDCA acts as an anti-inflammatory, cytoprotective, and anti-apoptotic signaling molecule [55]. Human interventional trials with healthy subjects supplementing their diet with UDCA, showed that the UDCA did not suppress endogenous BA synthesis. The fecal content of primary BA was stable, but researchers have recovered higher amounts of UDCA and its metabolite, LCA. Lithocholic acid is most likely formed through 7-dehydroxylation in colon lumen [56]. LCA is more hydrophobic than UDCA, thus it is thought to be more toxic. However, a recent publication by Lajczak-McGinley et al. claimed that both bile acids, UDCA and LCA, have anti-inflammatory and anti-apoptotic properties. In mouse model of intestinal inflammation (induced by dextran sulphate sodium in drinking water), administration of LCA and UDCA has reduced the gut-to-blood epithelial permeability and lowered mucosal myeloperoxidase activity in comparison to treatment with PBS (mock) or synthetic inactive bile acid. The authors have suggested that LCA acts by dampening inflammatory response in the colon [57].

Soon after the discovery of BA as agonists of FXR, a semi-synthetic ligand for FXR has been reported [58]. The compound is 6α-ethyl-3α,7α-dihydroxy-5-cholan-24-oic acid, also known as obeticholic acid (OCA). OCA is approximately 16- to 33-fold more potent than CDCA, a physiological FXR ligand [59]. It has been shown that OCA transactivates TGR5 as well, with an EC50 that is very close to that of LCA [60]. It is the first semi-synthetic bile acid that has been approved for use in humans. The first approved indication was the treatment of UDCA-non-responding primary biliary cholangitis. Currently, OCA is being tested in clinical trials in treatment of non-alcoholic steatohepatitis [61]. However, severe and adverse drug reactions, such as pruritus, gastrointestinal problems, increased risk of acute liver decompensation, and increased LDL cholesterol levels were reported [61,62].

## 6. Microbiome Profile and IBD

While most UC treatments target the immune system, a fecal microbiota transplantation (FMT) can induce remission in some patients. In patients who had received FMT and achieved remission, microbiome composition wasshifted towards colonization by *Eubacterium* and *Roseburia* species and enrichment of bile acid metabolism and short-chain fatty acids metabolism genes [63]. It is tempting to speculate that increased abundance of bacteria able to perform 7-β-dehydroxylation could increase anti-inflammatory action of UDCA. This idea is supported by positive results of fecal microbiome transplant with the 7-α-dehydroxylase-containing *Clostridium scindens*, which can dehydroxylate CA into DCA, and has achieved attenuation of colonization by *Clostridium difficile* in mice. It is further supported by restoration of secondary bile acid synthesis in colon of human patients recovered from recurrent *C. difficile* infection through FMT [64]. Short-chain fatty acids (acetate, propionate, and butyrate), which are mainly produced in the colon by anaerobic bacterial fermentation of dietary fiber, should be in the scope of IBD research as well. Butyrate decreases proinflammatory cytokine expression such as TNF-α via inhibition of NF-κB activation and IκBα degradation. It has been shown that TNF-α has an effect on activation of mucosal T helper-1 (Th1) cell immune responses [65]. Dietary fiber consumption may help to regulate Th1 immune responses in IBD. The presence of biofilms in UC patients, besides increased bile acids content, has been associated with a decrease in short-chain fatty acid producing bacterial genera, including *Faecalibacterium*, *Coprococcus*, *Subdoligranulum*, and *Blautia* [38]. Such intestinal biofilms were present in 57% of patients with irritable bowel syndrome [38]. *Faecalibacterium prausnitzii*, a known producer of anti-inflammatory short chain fatty acids, is highly sensitive to a slight increase in physiological concentrations of bile salts. Its growth is compromised by concentrations of 0.5% (wt/vol) [66].

From a clinical perspective, surgery is seen as a way to manage IBD, but it is irreversible. Ileocolonic resection or colectomy is typically reserved for individuals who have severe IBD and have exhausted other medical therapies. A recent study has investigated effects of surgery on the microbiome and metabolome in IBD patients [67]. Surgery and consecutive treatment influenced metabolite profiles of feces in post-surgery IBD patients. Cholic acid and chenodeoxycholic acid increased in subjects who underwent surgery, regardless of whether UC or CD was the reason. Notably, primary BA increased in CD patients with prior ileocolonic resection. In UC patients with colectomy and J pouch, a similar increase in primary bile acids was observed. Non-significant trends toward lower secondary bile acids in surgery samples were reported as well [67].

In patients after ileocolonic resection or colectomy, lower microbial abundance in fecal matter was observed regardless of the IBD type and resection localization. This reduction was attributed to the use of antibiotics. Changes in relative abundance of gut microbial communities were observed as well. Higher relative abundance of potential pathogens such as *Klebsiella pneumoniae*, *Enterococcus faecium*, and *Escherichia coli* was found in the surgery group compared with the non-surgery group. In contrast, butyrate-producing bacteria such as *Eubacterium rectale*, *E. eligens*, *F. prausnitzii*, and *Roseburia inulinivorans* and gut symbionts, including *Roseburia hominis* and *Ruminococcus obeum*, have had lower relative abundance in surgery samples than non-surgery samples. Enrichment analysis of metabolic pathways suggests the nitrate reduction to be significantly more abundant in surgery samples [67]. This is consistent with previous studies showing increased nitrate respiration in the inflamed gut [68]. In the light of previously discussed results on FTM in UC [63], reduced abundance of *Eubacterium* and *Roseburia* could suggest an unfavorable course of post-surgery IBD management. In another study on post-surgery microbiome, patients in remission hosted more *F. prausnitzii* and *Ruminococcus bromii*, whereas recurrence of CD was associated with increased colonization with α-/β-proteobacteria [69]. Moreover, enrichment in bacteria sharing specific enzymatic machinery (*Clostridium hylemonae*, *Ruminococcus gnavus* and *Enterococcus durans*) associated with the metabolism of bile acids (deconjugation, 7α-dehydroxylation) was observed [69]. In a study by Vila et al. on patients with CD, the removal of the ileocecal valve was associated with a reduction in microbiome richness and a decrease in metabolic pathways genes involved in the degradation of primary bile acids [70]. It is worth noting that the relative decrease of *F. prausnitzii* abundance was also associated with the surgery [70] as well as with a higher rate of IBD recurrence [71].

Interestingly, a recent case study reported a PSC patient on monotherapy with oral vancomycin, who achieved normalization of liver enzymes and resolution of UC symptoms with colonic mucosal healing. These improvements have persisted over 8 years, but the patient was sensitive to doses and vancomycin manufacturers’ brands [72]. Vancomycin is a glycopeptide antibiotic with bactericidal activity against Gram-positive bacteria. When administered orally, it has minimal systemic absorption; therefore, its effect presumably is confined to the intestinal lumen and perhaps mucosa. This suggests possible microbial dysbiosis as one of factors contributing to the development of colitis. In a small pilot clinical trial with PSC-IBD patients orally treated with vancomycin (*n* = 6), secondary bile acids have been significantly reduced during the 2-week-course of the treatment and for the first week after the vancomycin treatment ceased. These have returned to baseline values by third week after the vancomycin cessation. Only DCA concentration has changed significantly over the time and LCA concentrations demonstrated a non-significant decrease on vancomycin, with a somewhat delayed return to pre-vancomycin concentrations [73]. At baseline, there have been no significant differences between the overall fecal concentrations of BA in participants with IBD vs. PSC-IBD, or either group compared to healthy controls. Patients with IBD demonstrated a small, but significant, increase in LCA compared to those with PSC-IBD or to controls. Moreover, patients with PSC-IBD had a higher fraction of CA, as a percentage of total fecal BA, and thus a lower ratio of DCA:CA [73]. The latter result is in agreement with data reported by Torres et al. [37]. Oral vancomycin administration substantially decreased microbial diversity and post-vancomycin microbiota was assembled differently, with a relative increase in *Blautia* at the cost of *Bacteroides* [73].

## 7. Conclusions

In this review, current knowledge of bile acids and inflammatory bowel disease was described. The research results presented and reviewed here clearly indicate that physiological significance of BA reaches far beyond being emulsifiers helping in digestion of dietary lipids. BA should be also regarded as signaling molecules, involved in regulation of various physiological processes, including inflammation. A disturbed BA signaling seems to be associated with dysregulation of the mucosal immune system, compromised intestinal epithelial barrier, and an “unhealthy” gut microbiome. All of these may contribute to the pathogenesis of IBD. Restoration of proper BA signaling could be the basis of BA-mediated therapies in IBD. One way to reduce inflammation in IBD would be to make use of studies on BA metabolism and the gut microbiome. The combination of probiotics and FTM, which would enhance the production of beneficial secondary BA and other small compounds, seems to be an appealing approach. Another way would be the achievement of intestinal homeostasis by treatment with, yet-to-be-developed, agonists/antagonists of known nuclear and membrane BA receptors found in epithelial, hepatic or immune cells. This could have an impact beyond the treatment of IBD, due to involvement of FXR, and other receptors (PXR, CAR, VDR), in regulation of cholesterol metabolism. Thus, further advancements in the field of BA signaling are to be expected.

## Figures and Tables

**Table 1:** Relative composition of bile acids in gallbladder and feces.

	Gallbladder BA Composition [%]	Feces BA Composition [%]
CA (3α,7α,12α)	35	2
CDCA (3α,7α)	35	2
DCA (3α,12α)	25	34
LCA (3α)	1	29
UDCA (3α,7β)	2	2
other	2	31

Values from [11]. BA, bile acids; CA, cholic acid; CDCA, chenodeoxycholic acid; DCA, deoxycholic acid; LCA, lithocholic acid; UDCA, ursodeoxycholic acid. “Other” is a term for oxo-, 3β-hydroxy, and sulfo-derivatives of bile acids. Numbers in brackets indicate sites of hydroxylation of the steroid backbone.
